# HiCoDG: A Hierarchical Data-Gathering Scheme Using Cooperative Multiple Mobile Elements ^†^

**DOI:** 10.3390/s141224278

**Published:** 2014-12-17

**Authors:** Duc Van Le, Hoon Oh, Seokhoon Yoon

**Affiliations:** Department of Electrical and Computer Engineering, University of Ulsan, Ulsan 680-749, Korea; E-Mails: anhduc.mta@gmail.com (D.V.L.); hoonoh@ulsan.ac.kr (H.O.)

**Keywords:** mobile data gathering, multiple mobile elements, cooperative data gathering, optimal trajectories

## Abstract

In this paper, we study mobile element (ME)-based data-gathering schemes in wireless sensor networks. Due to the physical speed limits of mobile elements, the existing data-gathering schemes that use mobile elements can suffer from high data-gathering latency. In order to address this problem, this paper proposes a new hierarchical and cooperative data-gathering (HiCoDG) scheme that enables multiple mobile elements to cooperate with each other to collect and relay data. In HiCoDG, two types of mobile elements are used: the mobile collector (MC) and the mobile relay (MR). MCs collect data from sensors and forward them to the MR, which will deliver them to the sink. In this work, we also formulated an integer linear programming (ILP) optimization problem to find the optimal trajectories for MCs and the MR, such that the traveling distance of MEs is minimized. Two variants of HiCoDG, intermediate station (IS)-based and cooperative movement scheduling (CMS)-based, are proposed to facilitate cooperative data forwarding from MCs to the MR. An analytical model for estimating the average data-gathering latency in HiCoDG was also designed. Simulations were performed to compare the performance of the IS and CMS variants, as well as a multiple traveling salesman problem (mTSP)-based approach. The simulation results show that HiCoDG outperforms mTSP in terms of latency. The results also show that CMS can achieve the lowest latency with low energy consumption.

## 1. Introduction

Wireless sensor networks (WSNs) that consist of a number of low-cost, low-power stationary sensors have been widely used in various applications such as environmental monitoring, industrial sensing, and battlefield surveillance [1]. In WSNs, data gathering from distributed sensors deployed over a large area is one of the most important tasks. Data gathering in WSNs has traditionally been performed via multi-hop data forwarding to the sink. However, it is known that multi-hop forwarding leads to the energy-hole problem, where the energy of sensors near the sink is depleted quickly since they forward more data packets than sensors distant from the sink [2]. As a result, the energy-hole problem makes the network disconnected and decreases overall network lifetime.

In order to avoid the energy-hole problem, mobile data-gathering schemes have been studied [3–21]. In such schemes, mobile elements (MEs) (e.g., autonomous robots) are used to collect the data from sensors and bring the data to the sink. By using MEs, not only can the energy-hole effect be avoided but data gathering in a sparse or disconnected network also becomes possible because MEs can travel and directly collect the data from sensors. The drawback to this approach is long data-gathering latency since data delivery relies on the physical movements of MEs. In addition, high energy consumption by MEs is another issue to be addressed.

In order to achieve low data-gathering latency with minimal energy consumption, this paper proposes a new hierarchical and cooperative data-gathering (HiCoDG) scheme where two different types of MEs cooperate to collect and relay data from sensors to the sink. One type of ME is the mobile collector (MC), which collects data from sensors. Another type is the mobile relay (MR), which gathers data from MCs and delivers them to the sink. In HiCoDG, mobile collectors do not need to visit the sink, which can result in a significant improvement in terms of latency and energy consumption.

More specifically, in HiCoDG, sensors are first organized into one-hop clusters. The positions of clusterheads are considered *points of interest* in the network, which are partitioned into several groups with respect to geographic position. In each group, an MC is scheduled to periodically visit the points of interest, where it collects data from sensors within the cluster via one-hop communications. Then, MCs forward the data to the MR, which periodically travels from the sink to visit points of interest called meeting points (MPs) to receive data from MCs. Finally, the MR delivers the data when it returns to the sink.

In order to find optimum trajectories for MCs and the MR to minimize the total traveling distance, we define an optimization problem, named local data gathering with global relay (LDG-GR). Then, an integer linear programming (ILP) problem is formulated to find the solution to LDG-GR.

In this paper, depending on the way data are relayed from MCs to the MR, two variants of HiCoDG are considered: intermediate station (IS)-based and cooperative movement scheduling (CMS)-based schemes. In the IS scheme, each MC *drops off* data to an MP, which will store the data until the MR visits and *picks up* the data. In this case, special hardware (e.g., high-capacity data storage and batteries) is required for MPs. On the other hand, in the CMS scheme, the optimal movement speeds of MCs and the MR are predetermined, such that MCs and the MR can meet just in time during their journey to directly forward data using a wireless channel.

In addition, an analytical model was designed to estimate the average data-gathering latency in HiCoDG. The model can be useful for estimating whether MEs with a certain movement speed are able to achieve the required latency before they are actually deployed.

Extensive simulation and analysis were conducted to evaluate the performance of the two variants of HiCoDG. Those variants were also compared with multiple traveling salesman problem (mTSP)-based approach. The simulation results show that HiCoDG outperforms the mTSP-based approach in terms of latency and energy consumption. The simulation results also indicate that the CMS scheme can achieve the lowest latency.

The rest of the paper is organized as follows. In Section 2, we present related work and compare it to our algorithm. Section 3 presents the proposed scheme and algorithms in detail. In Section 4, an analytical model for estimating latency is described. Section 5 presents the simulation setup and performance analysis. Finally, Section 6 concludes the paper.

## 2. Related Work

In this section, we present an overview of previous studies on mobile data gathering, and compare those studies with our proposed algorithm.

There are a lot of recent studies on data gathering in WSNs using mobile elements. The majority of these studies considered the use of a single ME [5–12].

In these studies, an ME is scheduled to periodically visit sensors in the network and collect the data, then deliver them to the sink. For example, two studies [5,6] focused on the problem of finding the optimal trajectory and movement schedule for an ME, with the objective of maximizing network lifetime. More specifically, Wang *et al.* [5] assumed that the ME only visits some specific nodes in the network and stays for a given sojourn time to collect the data through multi-hop data transmissions. An linear programming (LP) optimization formulation was proposed to jointly determine the specific nodes the ME should visit and its sojourn time at each node, so that the network lifetime is maximized.

Gu *et al.* [8] and Somasundara *et al.* [9] proposed movement scheduling algorithms to schedule an ME such that the packet loss at sensors due to buffer overflow can be avoided. For example, Gu *et al.* [8] first organized the sensors in the network into a number of groups based on their data generation rates and locations. Then, the ME was scheduled to visit sensors in each group at an adequate frequency so the buffer overflow at the sensors is reduced.

In addition, Kumar *et al.* [11] and Sugihara and Gupta [12] focused on the problem of reducing data-gathering latency. Kumar *et al.* [11] proposed a single mobile collector-based data collection architecture that lowers latency by considering sensor clustering and long-range wireless communications. Sugihara and Gupta [12] proposed a heuristic scheduling algorithm that considers both sensor locations and time constraints to minimize latency in an 1D case problem.

Those studies differ from our work in that they use only one ME to collect the data from sensors. The potential drawback of this approach is less scalability. When the number of sensors or the network area increases, the path length the ME must travel increases accordingly. The ME will take a longer time to finish one round, which results in high data-gathering latency. Therefore, a single ME may not be sufficient for certain applications that require low latency in a large-scale WSN. In our work, we consider the use of multiple MEs for gathering data to reduce latency.

There have also been several studies that use multiple MEs for data gathering in WSNs [14–20]. For example, Zhao *et al.* [15] proposed a data-gathering scheme using multiple MEs that are controlled to move along parallel straight lines through the area of interest and collect data from sensors using multi-hop transmission. This scheme can work well in a large-scale WSN where the sensors are uniformly distributed. However, if not all of the sensors in the network are connected using a wireless link, the MEs may not cover some of them because they traverse only straight lines.

Kim *et al.* [16] defined two combinatorial optimization problems to find the trajectories, with the objective of minimizing data-gathering latency: the k-traveling salesperson problem with neighborhood (k-TSPN) and the k-rooted path cover problem with neighborhood (k-PCPN). They also proposed constant-factor approximation algorithms for those problems.

Those studies on multiple ME-based mobile data gathering differ from our work in that they schedule each ME to visit a part of the sensing field to collect data and all MEs need to return to the sink. This may lead to high data-gathering latency in a large-scale WSN where a number of sensors are deployed over a large area distant from the sink. In our scheme, sensors are first organized into groups, and a mobile collector (MC) that does not need to return to the sink is assigned to each group to collect data. The mobile relay is scheduled to periodically visit each group, receive the data from the MCs, and bring the data to the sink. Moreover, in our work, a cooperative movement scheduling algorithm is proposed to control the movements of the MCs and the MR in order to reduce the data-gathering latency in the network.

In addition, Aslanyan *et al.* [21] considered the use of multiple MEs that move with a random mobility pattern in the area of interest to collect the data, and deliver the collected data to the sink when they are close enough to the sink to ensure a direct transmission. Due to the random mobility, the data-gathering latency may not be estimated. In contrast, in our work, MEs are scheduled to move in cooperation to collect and relay the data to the sink. As a result, the average latency in our proposed schemes can be estimated, which is useful to determine whether or not the data-gathering scheme using MEs can satisfy the latency requirement before MEs are actually deployed.

## 3. Hierarchical and Cooperative Data Gathering (HiCoDG) Scheme

In this section, we present the proposed HiCoDG scheme in detail. The considered network consists of a number of stationary sensors that are deployed over an area of interest. Each sensor periodically generates data packets and saves them in its buffer. It is assumed that the positions of sensors are known a priori.

We consider the use of multiple mobile elements (e.g., autonomous robots equipped with RF transceivers) to gather data from sensors and to deliver the data to the sink located at a given position. More specifically, multiple mobile elements including mobile collectors and a mobile relay are deployed to cooperatively collect and relay the data.

**Figure 1.**
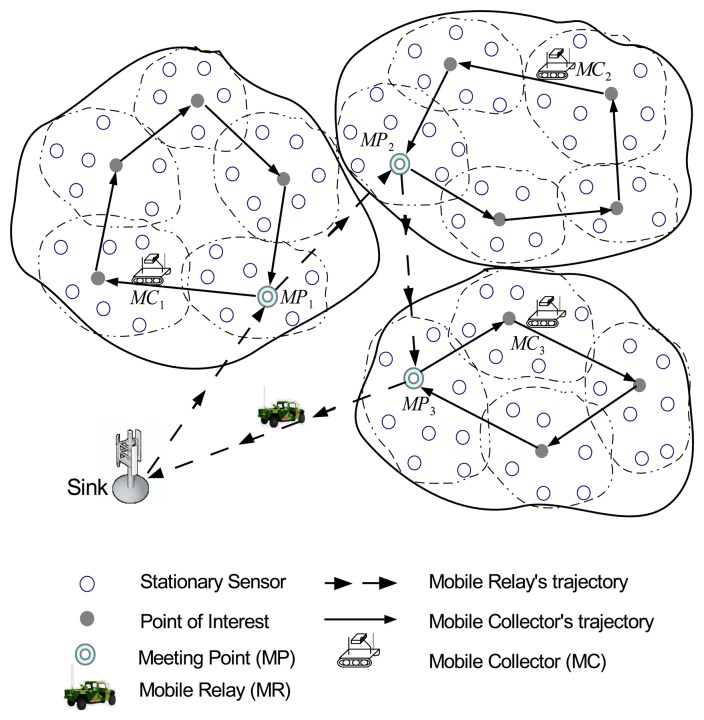
An overview of HiCoDG.

Figure 1 illustrates the overview of the proposed data-gathering scheme. In Figure 1, sensors are partitioned into three groups, each of which consists of several one-hop clusters. In each group, an MC moves along a closed path that consists of points of interest at which an MC collects data from sensors via one-hop wireless communications. The path of the MC begins and ends at an meeting point, which is one of the points of interest. The MC forwards the collected data to the MR via the MP or directly. The MR starts moving from the position of the sink, and visits each group at an MP and receives the data from the MP or MCs. It is assumed that MCs can be replaced with spare MCs in case they can not perform the mission due to power depletion or malfunction. In this paper, it is also assumed that collision between MEs can be avoided using existing collision avoidance algorithms [22,23], even if their movement paths intersect.

From here, we first describe the clustering algorithms that are used to partition sensors into several groups. Then, the optimization problem formulation to find the optimal trajectories for MEs is presented. Finally, two cooperative data relay schemes are described.

### 3.1. Clustering and Grouping Algorithms

In HiCoDG, sensor nodes are partitioned into *k* groups where *k* is the number of MCs used in the network. To form the groups, sensor nodes are first organized into one-hop clusters using a highest degree-based clustering algorithm [24]. More specifically, a node that has the maximum number of neighboring nodes is selected as the first clusterhead, and its neighboring nodes join the cluster. Among nodes that do not belong to the first cluster, another node with the maximum number of neighbors is selected to form the second cluster, and so on. This process is repeated until every node belongs to a cluster.

Then, the positions of clusterheads, which are regarded as points of interest (or points), are divided into *k* groups, each of which has an MC assigned to it. In this paper, we consider the use of two methods for partitioning the points into *k* groups: a K-means-based grouping algorithm and a sink position-based grouping (SPG) algorithm.

*K-means-based grouping algorithm*: We adopt K-means like a minimum mean distance algorithm (or K-means algorithm) [25], which uses the minimum mean distance between points as an objective function of grouping. More specifically, in the K-means algorithm, the points are divided into groups such that the mean distance between the group-head and members in each group is minimized.

*Sink position-based grouping (SPG) algorithm*: In order to achieve low data-gathering latency, it is desirable for the MR to travel a short tour so that it can deliver the data from the MCs to the sink in a short amount of time. However, when the K-means-based algorithm is used, some groups might be located far from the sink, which results in a long tour for the MR. Therefore, we propose the SPG algorithm. The main idea behind SPG is to divide the points into groups such that every group includes one point that is close to the sink. More specifically, in SPG, the area is first divided into *k* sub-parts such that the sink position is included in every sub-part. The points located in the same sub-part belong to the same group.

**Figure 2.**
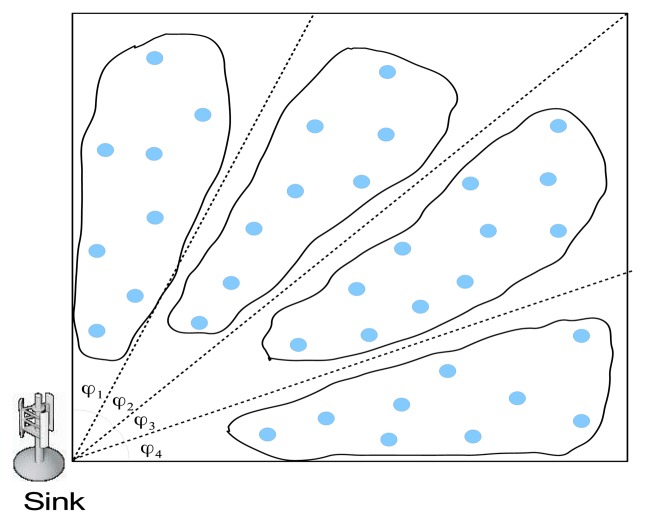
An example of sink position-based grouping.

Figure 2 illustrates grouping when SPG is applied. In Figure 2, the sink is located in the left corner of a rectangular area. Assume there are four MCs, *i.e.*, *k* = 4. Then, the area of interest is divided into four sub-parts represented by the dotted lines starting from the sink position such that *φ*_1_ = *φ*_2_ = *φ*_3_ = *φ*_4_ as shown in Figure 2.

### 3.2. Optimal Trajectory Formulation

Now, we consider the problem of finding the optimal trajectories for the MR and MCs that minimize latency and energy consumption in the network. Note that there have been a lot of studies on finding the optimal trajectory over visiting points in order to minimize total distance, such as the traveling salesman problem (TSP) [26], multiple salesman problem [27], and vehicle routing problem [28].

However, those studies cannot be used in our data-gathering architecture because they do not consider different types of MEs, the hierarchical architecture of MEs' movements, and the need for data exchange among MEs. More specifically, in our work, two types of mobile elements (MC and MR) are used. Each MC collects data from sensors in its group and forwards the data to the MR at a meeting point, which is one of the sensor positions in the group. The MR receives data from MCs at meeting points, and delivers the data to the sink.

Therefore, we define a new optimization problem.

#### Problem 1

Local data gathering with global relay (LDG-GR): Assume a network consists of a set of points *V* = {0, 1 … *N*}, where the element 0 represents the sink. *V* is partitioned into *k* + 1 subsets, *V*_0_, *V*_1_…*V_k_* where *V*_0_ = {0}. Using *k* MCs, and one MR, find the minimum total path length of the MCs and the MR. *MC_i_* (1 ≤ *i* ≤ *k*) must visit every point in *V_i_* once and come back to its starting point. The MR must visit at least one point in each *V_i_* and come back to its starting position *i.e.*, point 0 in *V*_0_.

#### Proposition 1

*LDG-GR is NP-complete*.

##### Proof

It can be readily shown that a Euclidean TSP [29] is polynomial-time reducible to LDG-GR, *i.e.*, Euclidean TSP ≤*_p_* LDG-GR. Suppose that a Euclidean TSP instance consists of a set of *k* “cities” *c*_1_, …, *c_k_*, a distance *d*(*i*, *j*) between every pair of cities *c_i_* and *c_j_* (1 ≤ *i*, *j* ≤ *k*), and an integer *D*. The corresponding LDG-GR instance has *k* subsets, *V*_1_, …, *V_k_*, each of which has only one element *i.e.*, |*V_i_*| = 1. Let *V_i_* have a point *i* as an element that corresponds to *c_i_*, and define the distance between a pair of points in $\cup_{i}^{k}V_{i}$ the same as *d*(*i*, *j*). Let point 0 be the sink. The instance of LDG-GR can be constructed in polynomial time. Note that in the constructed LDG-GR instance, only the MR has a traveling distances since every subset has only one point. Then, it is clear that if the length of the tour is *D* in Euclidean TSP, the total path length in LDG-GR becomes *D*. Conversely, if the total path length in LDG-GR is *D*, the length of the tour becomes *D* in the Euclidean TSP. LDG-GR is in NP, since a guessed path, obtained by using a non-deterministic algorithm, can be checked in polynomial time as to whether it has a total length of *D* and if every point is visited.

In this paper, we propose ILP formulation for jointly finding the optimal trajectories for both MCs and the MR. We model the network as a complete directed graph *G* = (*V*, *E*) where *E* = {(*i*, *j*) : *i*, *j* ∈ *V*, *i* ≠ *j*} is the set of arcs. Let *c_ij_* denote the travel cost (Euclidean distance) from point *i* to point *j*. We also define *x_ij_* as a binary variable that becomes 1 if *arc*(*i*, *j*) is on the trajectories of the MCs, but becomes 0 otherwise. Similarly, a binary variable *y_ij_* represents whether or not *arc*(*i*, *j*) is on the trajectory of the MR. We denote *n_i_* as the number of points in subset *V_i_ i.e.*, | *V_i_* |= *n_i_*. Note that *n*_0_ = 1. Also, *u_i_* is defined as the number of points that have been visited by the MCs and the MR up to point *i*. We also define $p_{i}^{\text{org}}\in V_{i}$ as the origin point of the trajectory solution of *MC_i_*. Note that $p_{i}^{\text{org}}$ can be an arbitrary point in *V_i_* and can be chosen a priori.

Then, the ILP problem can be formulated as follows:

(1) $$\text{minimize}\;\left(\overset{N}{\underset{i=1}{\text{∑}}}\overset{N}{\underset{j=1}{\text{∑}}}c_{ij}x_{ij}+\overset{N}{\underset{i=0}{\text{∑}}}\overset{N}{\underset{j=0}{\text{∑}}}c_{ij}y_{ij}\right)$$

subject to:

(2) $$\begin{array}{cc} \overset{N}{\underset{i=1,i\neq j}{\text{∑}}}x_{ij}=1, & j=1\ldots N \end{array}$$

(3) $$\begin{array}{cc} \overset{N}{\underset{j=1,j\neq i}{\text{∑}}}x_{ij}=1, & i=1\ldots N \end{array}$$

(4) $$\begin{array}{cc} \underset{i\in V_{l}}{\text{∑}}\underset{j\in V\backslash V_{l}\cup V_{0}}{\text{∑}}x_{ij}=0, & l=1\ldots k \end{array}$$

(5) $$\begin{array}{cc} u_{p_{l}^{\text{org}}}=1, & l=1\ldots k \end{array}$$

(6) $$\begin{array}{l} u_{i}-u_{j}+1\leq \left(n_{l}-1\right)\left(1-x_{ij}\right)\\ i,j\in V_{l}\backslash \{p_{l}^{\text{org}}\},l=1\ldots k \end{array}$$

(7) $$\begin{array}{cc} \underset{i\in V_{l}}{\text{∑}}\underset{j\in V\backslash V_{l}}{\text{∑}}y_{ij}=1, & l=1\ldots k \end{array}$$

(8) $$\begin{array}{cc} \underset{i\in V\backslash V_{l}}{\text{∑}}y_{ij}=\underset{i\in V\backslash V_{l}}{\text{∑}}y_{ji}, & j\in V_{l},l=1\ldots k \end{array}$$

(9) $$u_{0}=1$$

(10) $$\begin{array}{cc} u_{i}-u_{j}+1\leq \left(N-1\right)\;\left(1-y_{ij}\right), & i,j\in V\backslash V_{0} \end{array}$$

The objective function in Equation (1) is for minimizing the total distance traveled by both the MCs and the MR. Equations (2)–(6) define constraints on the optimal trajectories of the MCs, whereas Equations (7)–(10) represent constraints on the optimal trajectory of the MR. More specifically, constraints Equations (2) and (3) ensure that every point except 0 is visited exactly once by only one MC. Constraint Equation (4) states that MCs do not travel between two points that belong to two different subsets (*i.e.*, groups). Constraints Equations (5) and (6) eliminate the subtours in the trajectories of the MCs in a way similar to the MTZ subtour elimination of the TSP [26].

On the other hand, constraint Equation (7) ensures that the MR visits each group exactly once at one point. Note that among all points of interest, the point that will be visited by the MR is called a meeting point (MP). Constraint Equation (8) ensures that the MR enters and leaves each group at the same MP. Note that *MP_i_* may be different from $p_{i}^{\text{org}}$ which was arbitrarily chosen to find the optimal trajectories. Constraints Equations (9) and (10) eliminate the subtour in the trajectory of MR.

Note that, in case there are constraints (e.g., obstacles) on the link between two points, the link cost value can be set to a high value (or even an infinite number). Due to the objective function that minimizes the path cost, such high cost links will be avoided in the solution. In other words, the ILP formulation can find the solution even when there are physical constraints on the links.

### 3.3. Cooperative Data Relay Scheme

Along the optimal trajectories obtained from the ILP formulation, each MC periodically performs data gathering in its group. On each tour, an MC starts from an MP, visits all points in the group, and returns to the MP. The MR also periodically travels to receive data from the MCs at the MPs. In order for the MR to relay the data from MCs to the sink, we consider two schemes: IS-based data relay and cooperative movement scheduling CMS-based data relay. In the IS scheme, MPs require more powerful HW than regular points of interest, while CMS does not require special HW.

**Figure 3.**
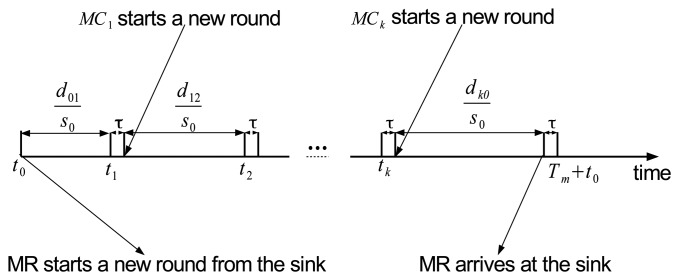
Cooperative movement time scheduling.

(1) IS-based data relay
  In this scheme, it is assumed that the points that are selected as MPs are equipped with high-capacity data storage and high-capacity batteries. During its journey, an MC sends its collected data to its MP when they can establish a direct wireless communications channel. Then, the MP stores the data and forwards them when the MR visits. The MR receives data from all MPs and returns to the sink.
  Note that, in the IS scheme, MCs and the MR move independently. Due to lack of cooperation among nodes, the MR can visit an MP before an MC sends data to that MP. In that case, the MR cannot receive any data from the MP until the next visit. As a result, the MR wastes energy, and data-gathering latency may increase. Moreover, the need for data storage and high-capacity batteries at MPs increases system cost.
  In order to address these problems, we propose a CMS-based data relay algorithm, where the movements of MCs and the MR are scheduled such that the MR can directly receive the collected data from MCs while the MR and MCs are traveling.
(2) CMS-based data relay
  The main idea behind CMS is that the movement speeds of MCs and the MR are controlled so that MCs can meet the MR at MPs during the periodic movements and can forward data to the MR using a direct wireless channel. Let *L_i_* (*i* = 0 … *k*) denote the path (or tour) length of each ME (MC or MR), where *L*_0_ is the tour length of the MR and *L_i_* (1 ≤ *i* < *k*) the tour length of *MC_i_*. We also define *s_max_* as the maximum movement speed of MEs.
  Recall that the path of the MR consists of *k* + 1 meeting points, *MP_i_* (0 ≤ *i* ≤ *k*) where *MP*_0_ is the sink's position. Let *τ* denote the time duration in which the MR stays to receive the data from *MC_i_* at *MP_i_*(*i* ≠ 0), or to send the data to the sink at *MP*_0_. We also define *T_m_* as the time it takes for the MR to finish one tour, which includes moving time and sojourn times at MPs. Then, the value of *T_m_* can be calculated as
  (11) $$T_{m}=\frac{L_{\text{max}}}{s_{\text{max}}}+\left(k+1\right)\tau$$
  where *L_max_* = max(*L*_0_, *L*_1_, …, *L_k_*).
  The speeds of MEs are determined such that the MR can periodically meet *MC_i_* at every *T_m_*. Using *T_m_* and the tour length of each ME, the speed of each ME can be calculated as follows:
  (12) $$\{\begin{array}{l} s_{0}=\frac{L_{0}}{T_{m}-\left(k+1\right)\tau }\\ \begin{array}{cc} s_{i}=\frac{L_{i}}{T_{m}-\tau }, & 1\leq i\leq k \end{array} \end{array}$$
  where *s*_0_ is the speed of the MR and *s_i_* is the speed of *MC_i_*. Note that *s*_0_ and *s_i_* are equal to or less than *s_max_*.
  Also, denote *t*_0_ as the time at which the MR leaves the sink and *d_ij_* as the distance from *MP_i_* to *MP_j_*. Then, during the same tour, the MR meets *MC_i_* at time *t_i_*(*i* ≠ 0), which can be calculated as
  (13) $$t_{i}=t_{0}+\left(i-1\right)\tau +\frac{1}{s_{0}}\overset{i}{\underset{j=1}{\text{∑}}}d_{\left(j-1\right)j}$$
  In CMS, the MR starts a new tour at the sink at every *T_m_* as shown in Figure 3. The MR begins its journey from *MP*_0_, and visits *MP_i_*, where it meets *MC_i_* and stays for *τ* seconds to receive data. Then, the MR returns to *MP*_0_, stays for *τ* seconds to deliver data to the sink, and then starts another journey. Note that it is assumed that the initial positions of *MC_i_* (1 ≤ *i* ≤ *k*) are also determined such that *MC_i_* can meet the MR at *t_i_*. After sending the data to MR, *MC_i_* starts new round at *t_i_* + *τ* as shown in Figure 3. By using the speed from Equation (12), *MC_i_* will periodically meet the MR every *T_m_* seconds during future rounds.

## 4. Analytical Model for Estimating Data Gathering Latency in HiCoDG

In wireless sensor networks, the application often has a specific requirement for data-gathering latency. Therefore, it is important and necessary to estimate whether or not the mobile data-gathering system using MEs can satisfy the latency requirement before the MEs are actually deployed. In order to address this issue, in this section, we discuss an analytical model to estimate data-gathering latency in the proposed HiCoDG.

We first present some general assumptions for analysis. Then, an analytical model to estimate data-gathering latency is described. Finally, we verify the model by comparison with simulation results.

### 4.1. General Assumptions

We consider a network consisting of *k* + 1 MEs, including *k* MCs and one MR. The points of interest (or points) in the network are partitioned into *k* groups *G_i_* (*i* = 1, …, *k*). *MC_i_* visits and collects data at points in group *G_i_*. Recall that *L*_0_ and *L_i_* (*i* = 1, …, *k*) denote the path lengths of the MR and *MC_i_*, respectively. Similarly, we define *T_i_* (*i* = 0, …, *k*) as the time that it takes for MR and *MC_i_* to finish one tour, respectively. In addition, recall that *s*_0_ and *s_i_* are movement speeds of the MR and *MC_i_*, respectively. Without loss of generality, in the model, we do not consider latency of wireless communications since the network transmission speed is much higher than the movement speed of MEs. Thus, we can have

(14) $$\{\begin{array}{l} T_{0}=\frac{L_{0}}{s_{0}}\\ \begin{array}{cc} T_{i}=\frac{L_{i}}{s_{i}} & \left(\text{i}=1,\ldots ,\text{k}\right) \end{array} \end{array}$$

It is assumed that at every point on the paths of the MCs, a data packet is periodically generated at the rate of *R* (in packets per second). The packet is stored in the buffer of the point until an MC collects it. We also assume that on the path of each MEs (the MR or MCs), the points are randomly placed in an uniform distribution.

### 4.2. Estimation of Average Data-Gathering Latency

**Figure 4.**
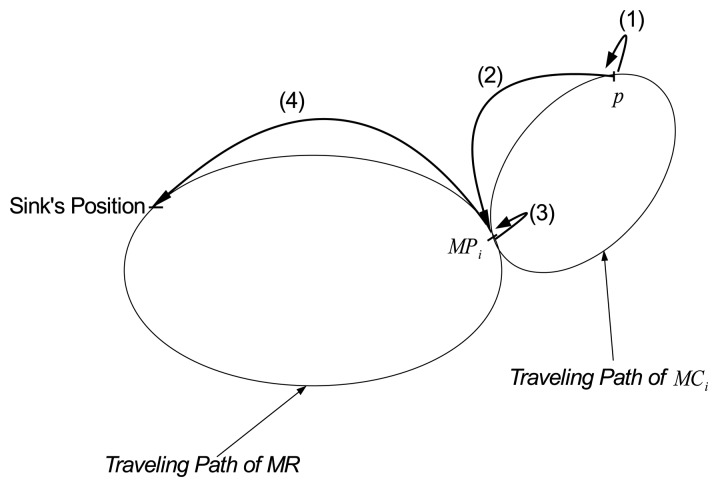
Time components of data latency.

In the model, we estimate the average data-gathering latency of a packet generated at an arbitrary point *p* located on the path of *MC_i_* in group *G_i_*. In HiCoDG, the latency of the packet at point *p* is the sum of four time components as shown in Figure 4, which are:

(1) the time duration for the packet to wait in the buffer at point *p* from its generation until *MC_i_* collects it.
(2) the time taken for *MC_i_*, which carries the packet, to travel from point *p* to meeting point *MP_i_*.
(3) the time duration for the packet to wait in *MP_i_* until the *MR* picks it up.
(4) the time taken for the *MR* to travel from *MP_i_* to the sink.

From here, we present the estimation of each of the above time components to obtain the average data-gathering latency of the packet collected by *MC_i_* at point *p*.

#### 4.2.1. Estimation of Waiting Time in the Buffer

We first estimate the expected latency for a packet waiting in the buffer at point *p*. Let *M* denote the total number of packets that *MC_i_* collects at point *p* at each arrival. Since *MC_i_* periodically visits and collects the packets from point *p* at every interval *T_i_*, the value of *M* is the total number of packets generated at point *p* during period *T_i_*. Then, we have *M* ≈ *RT_i_*, assuming $T_{i}\gg \frac{1}{R}$.

**Figure 5.**
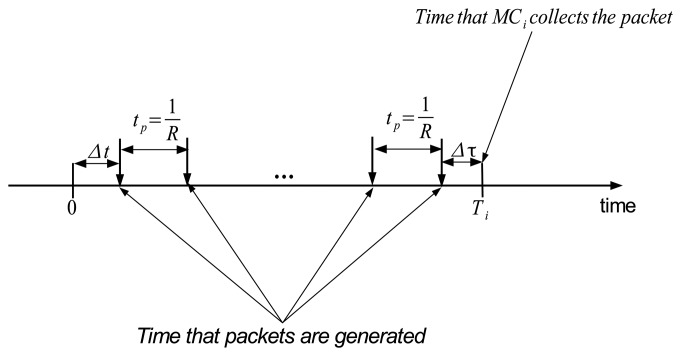
Estimation of the average waiting time of a packet in the buffer.

We suppose that packets are generated in the time interval [0, *T_i_*]. Then, those packets will be collected by *MC_i_* at *T_i_* as shown in Figure 5. Let Δ*t* denote the time that the first packet is generated at point *p* during *T_i_*, and let Δ*τ* denote the time difference between the time the last packet is generated and *T_i_*. If we define ϒ as the total waiting time of *M* packets in the buffer, then the value of ϒ can be simply calculated as

(15) $$ϒ=\overset{M}{\underset{j=1}{\text{∑}}}\frac{j}{R}+M\Delta \tau =\frac{M\left(M+1\right)}{2R}+M\Delta \tau$$

Let *t_p_* denote the packet interval and $t_{p}=\frac{1}{R}$. Then, we have

(16) $$\Delta \tau =T_{i}-\left(\;\left\lfloor \frac{T_{i}}{t_{p}}\right\rfloor \;t_{p}+\Delta t\right)$$

By substituting Δ*τ* into Equation (15), we obtain

(17) $$ϒ=\frac{t_{p}M\left(M+1\right)}{2}+M\;\left(T_{i}-\left\lfloor \frac{T_{i}}{t_{p}}\right\rfloor \;t_{p}-\Delta t\right)$$

Let $t_{b}^{i}$ denote the mean time of a packet waiting in the buffer at point *p*. Then,

(18) $$t_{b}^{i}=\frac{ϒ}{M}=\frac{t_{p}\left(M+1\right)}{2}+T_{i}-\left\lfloor \frac{T_{i}}{t_{p}}\right\rfloor \;t_{p}-\Delta t$$

By substituting $M=RT_{i}=\frac{T_{i}}{t_{p}}$ into Equation (18), we get

(19) $$t_{b}^{i}=\frac{3T_{i}}{2}-\left\lfloor \frac{T_{i}}{t_{p}}\right\rfloor \;t_{p}+\frac{t_{p}}{2}-\Delta t$$

Since the first packet can be generated in a random time, Δ*t* can be considered a uniform random variable that takes the value in [0, *t_p_*). As a result, $t_{b}^{i}$ is also a random variable because it is a function of Δ*t*. Thus, the expected value of $t_{b}^{i}$ can be expressed as

(20) $$E\left[t_{b}^{i}\right]=\frac{1}{t_{p}}\int_{0}^{t_{p}}\left(\frac{3T_{i}}{2}-\left\lfloor \frac{T_{i}}{t_{p}}\right\rfloor \;t_{p}+\frac{t_{p}}{2}-t\right)dt=\frac{3T_{i}}{2}-\left\lfloor \frac{T_{i}}{t_{p}}\right\rfloor \;t_{p}$$

#### 4.2.2. Estimation of Traveling Time in *MC_i_*

After *MC_i_* collects the packet from point *p*, it will bring the packet to *MP_i_*. Recall that all points on the path of *MC_i_* are randomly placed in a uniform distribution. Let *χ* be a random variable that represents the traveling distance from *MP_i_* to arbitrary point *p* on the path of *MC_i_*. Then, the probability density function (pdf) of *χ*, *f_χ_*(*x*), is expressed as

(21) $$f_{\chi }\left(x\right)=\{\begin{array}{ll} \frac{1}{L_{i}} & \text{if}\;x\in [0,L_{i})\\ 0 & \text{o}/\text{w} \end{array}$$

Let $t_{mc}^{i}$ denote the time the packet is in *MC_i_* before *MC_i_* arrives at *MP_i_* at the end of its round. Then, $t_{mc}^{i}$ is the time it takes for *MC_i_* to travel from point *p* to *MP_i_*, which is

(22) $$t_{mc}^{i}=T_{i}-\frac{\chi }{s_{i}}$$

Since $t_{mc}^{i}$ is a function of the random variable *χ*, the expected value of $t_{mc}^{i}$ can be calculated as

(23) $$E\left[t_{mc}^{i}\right]=\frac{1}{L_{i}}\int_{0}^{L_{i}}(T_{i}-\frac{x}{s_{i}})dx=T_{i}-\frac{L_{i}}{2s_{i}}=\frac{T_{i}}{2}$$

note that $T_{i}=\frac{L_{i}}{s_{i}}$

#### 4.2.3. Estimation of Waiting Time in *MP_i_*

In the IS scheme, the packet collected by *MC_i_* is stored in *MP_i_* until the MR arrives and picks it up. Now we estimate the time that the packet might wait at *MP_i_* for the MR under the IS scheme. Note that in CMS, since MR and *MC_i_* are scheduled to periodically meet each other at *MP_i_*, the time a packet waits in *MP_i_* equals zero.

Recall that *MR* and *MC_i_* periodically visit *MP_i_* every *T*_0_ and *T_i_*(1 ≤ *i* ≤ *k*), respectively. We define {*Y*_1_, *Y*_2_, …, *Y_l_*, …} as the time that *MC_i_* arrives at *MP_i_* during its journey, where *Y_l_* is the time of the *l^th^* arrival. Note that *Y_l_*_+1_ − *Y_l_* = *T_i_*. Also, let {*Z*_1_, *Z*_2_, …, *Z_h_*, …} denote the time that the *MR* visits *MP_i_*, where *Z_h_* is the time that MR arrives at *MP_i_* in its *h^th^* round.

**Figure 6.**
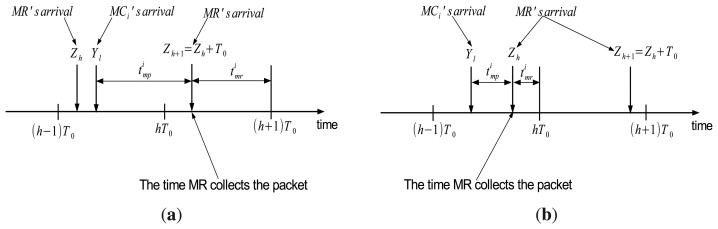
Estimating the waiting time of a packet in the MP. (**a**) Case 1: *Z_h_* < *Y_l_*; (**b**) Case 2: *Z_h_* ≥ *Y_l_*.

Suppose that *MC_i_* collects the packet at point *p* in the *l^th^* round and brings the packet to *MP_i_* at time *Y_l_* (*l* ≥ 1). Also, assume that *MC_i_* arrives at *MP_i_* in the interval [ (*h* – 1)*T*_0_, *hT*_0_], *i.e.*, *Y_l_* ∈ [ (*h* – 1)*T*_0_, *hT*_0_] as shown in Figure 6. In the time interval [ (*h* – 1)*T*_0_, *hT*_0_], the MR starts its *h^th^* round from the sink's position at (*h* – 1)*T*_0_, visits *MP_i_* at *Z_h_*, and finally returns to the sink at *hT*_0_.

Since *MP_i_* is considered as an arbitrary point randomly placed in the path of the *MR*, *Z_h_* is a uniform random variable that represents the time the *MR* arrives at *MP_i_* in the interval [ (*h* – 1)*T*_0_, *hT*_0_]. Thus, the pdf of *Z_h_* can be expressed as

(24) $$f_{Z}\left(z\right)=\{\begin{array}{ll} \frac{1}{T_{0}} & \text{if}\;z\in \left(\left(h-1\right)T_{0},hT_{0}\right)\\ 0 & \text{o}/\text{w} \end{array}$$

In the interval [ (*h* – 1)*T*_0_, *hT*_0_], the packet stored in *MP_i_* at time *Y_i_* is collected by the *MR* at time *Z_h_* if the MR visits *MP_i_* after the time *MC_i_* arrives *i.e.*, *Z_h_* ≥ *Y_l_*. Otherwise, the *MR* will collect the packet during its next visit at time *Z_h_*_+1_ in the interval [*hT*_0_, (*h* + 1)*T*_0_]. Note that *Z_h_*_+1_ = *Z_h_* + *T*_0_. Two cases for this collection are shown in Figure 6.

We define $t_{mp}^{i}$ as the waiting time of the packet in *MP_i_*. Then, the value of $t_{mp}^{i}$ is calculated as shown in Figure 6, which is

(25) $$t_{mp}^{i}=\{\begin{array}{ll} Z_{h}+T_{0}-Y_{l} & \text{if}\;Z_{h}<Y_{l}\\ Z_{h}-Y_{l} & \text{o}/\text{w} \end{array}$$

Since $t_{mp}^{i}$ is a function of the random variable *Z_h_*, it is also a random variable. Thus, the expected value of $t_{mp}^{i}$ can be calculated as

(26) $$E\left[t_{mp}^{i}\right]=\frac{1}{T_{0}}\left(\int_{(h-1)T_{0}}^{Y_{l}}\left(z+T_{0}-Y_{l}\right)dz+\int_{Y_{l}}^{hT_{0}}\left(z-Y_{l}\right)dz\right)=\frac{T_{0}}{2}$$

#### 4.2.4. Estimation of Time in MR

After the MR collects the packet at *MP_i_*, it brings the packet to the sink. Let $t_{mr}^{i}$ denote the time it takes for the MR to move from *MP_i_* to the sink's position. That is

(27) $$t_{mr}^{i}=\{\begin{array}{ll} (h+1)T_{0}-Z_{h+1} & \text{if}\;Z_{h}<Y_{l}\\ hT_{0}-Z_{h} & \text{o}/\text{w} \end{array}$$

Since *Z_h_*_+1_ *= Z_h_* + *T*_0_, the value of $t_{mr}^{i}$ is rewritten as

(28) $$t_{mr}^{i}=hT_{0}-Z_{h}$$

Similarly, the expected value of $t_{mr}^{i}$ can be calculated as

(29) $$E\left[t_{mr}^{i}\right]=\frac{1}{T_{0}}\int_{(h-1)T_{0}}^{hT_{0}}(hT_{0}-z)dz=\frac{T_{0}}{2}$$

Define $t_{\text{mean}}^{i}$ as the mean latency of a packet collected by *MC_i_* from arbitrary point *p* in group *G_i_*. From Equations (20), (23), (26) and (29), the value of $t_{\text{mean}}^{i}$ can be approximated as

(30) $$t_{\text{mean}}^{i}=\{\begin{array}{ll} E\left[t_{b}^{i}\right]+E\left[t_{mc}^{i}\right]+E\left[t_{mp}^{i}\right]+E\left[t_{mr}^{i}\right]=2T_{i}-\left\lfloor \frac{T_{i}}{t_{p}}\right\rfloor \;t_{p}+T_{0} & \text{if IS is used}\\ E\left[t_{b}^{i}\right]+E\left[t_{mc}^{i}\right]+E\left[t_{mr}^{i}\right]=2T_{i}-\left\lfloor \frac{T_{i}}{t_{p}}\right\rfloor t_{p}+\frac{T_{0}}{2} & \text{if CMS is used} \end{array}$$

Note that in the IS scheme, the packet may need to wait at the MP until the MR visits the MP and picks it up. Thus, the latency of the packet under IS consists of the time the packet waits in the MP In contrast, under CMS, the packet is immediately delivered to the MR when the *MC* arrives at the MP since the MR and MC are scheduled to periodically meet each other at the MP.

By substituting $T_{i}=\frac{L_{i}}{s_{i}}$ and $T_{0}=\frac{L_{0}}{s_{0}}$ into Equation (30), we obtain

(31) $$t_{\text{mean}}^{i}=\{\begin{array}{ll} \frac{2L_{i}}{s_{i}}-\left\lfloor \frac{L_{i}}{s_{i}t_{p}}\right\rfloor \;t_{p}+\frac{L_{0}}{s_{0}} & \text{if IS is used}\\ \frac{2L_{i}}{s_{i}}-\left\lfloor \frac{L_{i}}{s_{i}t_{p}}\right\rfloor \;t_{p}+\frac{L_{0}}{2s_{0}} & \text{if CMS is used} \end{array}$$

Note that in Equation (31), the movement speeds *s_i_* of MEs in two cases for IS and CMS may have different values from each other.

We also estimate the movement energy consumption of MEs for data gathering. Let II denote the total energy consumed by MEs for their movement during data collection time *t_c_*. The value of II can be calculated as

(32) $$\Pi =\rho \left(t_{c}s_{0}+\overset{k}{\underset{i=1}{\text{∑}}}t_{c}s_{i}\right)$$

where *ρ* represents the energy consumed to travel a meter, and we assume *ρ* = 8.27 joules.

### 4.3. Result Analysis

Equation (31) gives the estimated mean latency of a packet collected by *MC_i_*. Now, in order to verify the accuracy of the analytical model, we compare the results from the model with the results from simulations using a simulator developed in Matlab. For comparison, we consider a network that consists of one MR and four MCs. The MR and MCs have a given path length. There are 100 points randomly placed on the path of each MC. Each point periodically generates a packet every 1 s, *i.e.*, the data generation rate *R* = 1 (packet/second). Also, we assume the MEs have maximum movement speed *s_max_*. In CMS, the movement speeds of the MR and MCs are determined using Equations (11) and (12). In IS, every ME moves at the same speed.

We collect the average latency of the packet from the simulation and the analytical model with two different path length configurations for MEs, as shown in Table 1. For each path length configuration for MEs, we compare the latency under CMS and IS. All MEs in IS move at the average movement speed for MEs in CMS.

**Figure 7.**
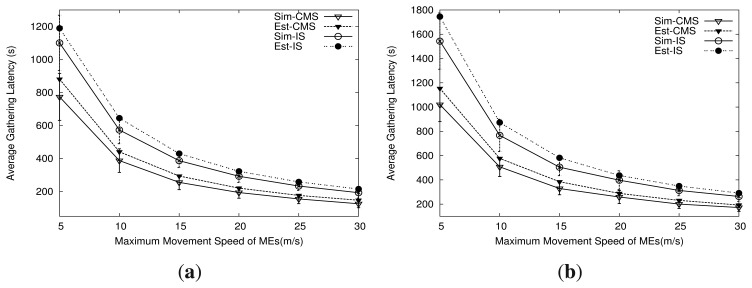
Average data gathering latency from estimation and simulation (mean ± standard deviation). (**a**) Configuration 1; (**b**) Configuration 2.

Figure 7 shows the average latency of the packet from the estimation and the simulation over variations in maximum movement speeds for MEs. More specifically, Figure 7 show the results for path length configurations 1 and 2, respectively Each result from the simulation is the average value over 100 simulation runs. In each simulation run, the positions of points on the path for the MEs are randomly chosen. Also, the result from the simulation is the average latency of all packets collected at the sink during the simulation time of 3000 s. In the analytical model, the result is the average of the estimated latency of the packets collected by four MCs.

As shown in Figure 7, the average latency estimated from the analytical model is close to the average latency from the simulation in the two different path length configurations. The gap between the results from the model and the simulation is narrow and the model consistently has a higher latency than the simulation. In other words, the analytical model can provide an upper bound for average latency compared with the simulation.

We also collect energy consumption by the MEs for movement. Table 2 shows the total energy consumed by MEs' movements during simulation time with two different path length configurations. As shown in Table 2, for each configuration, both CMS and IS show the same movement energy consumption, since they have the same average speed of MEs. Note that for each data relay scheme, the analytical model and the simulation also have the same result of energy consumption.

Moreover, the results shown in Figure 7 and Table 2 indicate that on a given amount of energy consumed for the movement of MEs, CMS achieves lower latency than IS. This confirms that the movement scheduling scheme can reduce the data gathering latency.

In summary, in this section we present an analytical model for approximating average latency of packets collected by MEs in HiCoDG, which is verified by comparison with the simulation results. Note that the model is useful for assessing whether MEs can provide the required latency before they are deployed.

## 5. Performance Evaluation

In this section, we evaluate the performance of HiCoDG variants by comparing them with an mTSP-based approach. Recall that in HiCoDG, two cooperative data relay schemes are proposed, IS and CMS. Also, two different grouping algorithms (*i.e.*, K-means and SPG) are considered. Therefore, four combinations of HiCoDG variants are evaluated in the simulations.

- IS with K-means
- IS with SPG
- CMS with K-means
- CMS with SPG

We first present the simulation parameters and performance metrics, and then discuss the performance results.

### 5.1. Simulation Setup

Recall that in HiCoDG, MCs visit a point of interest (*i.e.*, a clusterhead) and collect the data from the clusterhead, which received them from sensors within its domain. In this section, for the sake of brevity, we consider a simplified network configuration that only includes a certain number of points (*i.e.*, clusterheads) that are assumed to generate data for their cluster.

The considered network consists of 32 points of interest (POIs) that are randomly deployed in an area of 2000 m × 2000 m. Each POI generates a data packet every 1 s and stores the packet in its buffer until an MC collects it. One MR collects data from four MCs and delivers the data to the sink (*i.e.*, five MEs are used). Accordingly, POIs are divided into four groups.

In the mTSP-based approach, we use the ILP formulation for the mTSP in [27] to find *m* optimal trajectories that begin and end at the position of the sink with the objective of minimizing the total distance traveled. One ME is assigned to each trajectory. Also under mTSP, five MEs (*i.e.*, *m* = 5) are arranged on five routes to visit POIs and return to the sink.

To simulate data gathering in wireless sensor networks, we use a network simulator, Qualnet 6.1, in which the controllable mobility simulator architecture, ConMoSim [30] was integrated to simulate the mobility of MEs. The GNU Linear Programming Kit [31] was used to solve the ILP problem of finding optimal trajectories for MEs in both HiCoDG and mTSP.

Four performance metrics are used to evaluate the performance of algorithms:

- *maximum gathering latency*: the maximum value over elapsed times for all packets from generation to arrival at the sink.
- *average gathering latency*: the average value over elapsed times for all packets from generation to arrival at the sink.
- *movement energy consumption*: the total energy consumed by MEs' movements.
- *maximum number of packets in buffer of ME*: the maximum number of packets that is stored in the buffer of mobile elements.

Also, the simulation time is 10,000 s, and we assume that an ME consumes 8.27 joules to travel one meter (or 0.21 J/inch) [32].

### 5.2. Performance Analysis

**Figure 8.**
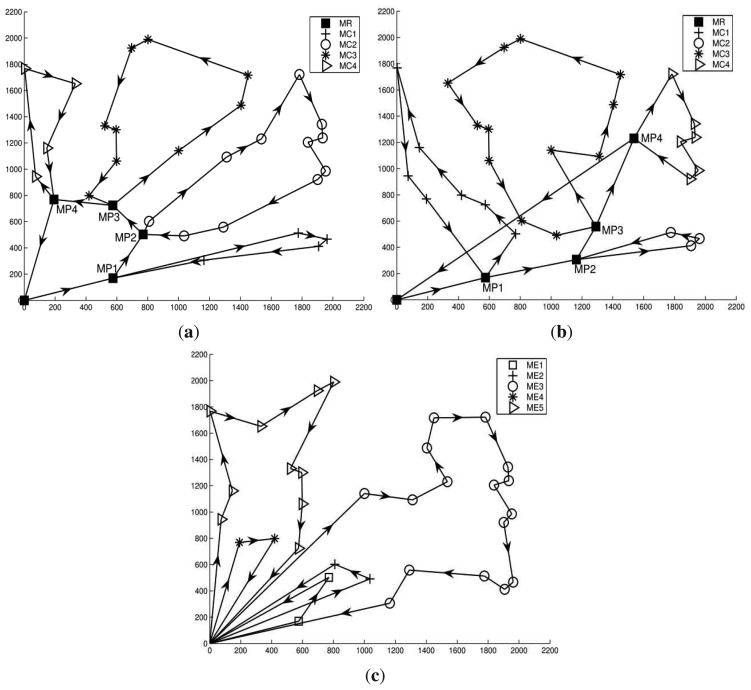
Movement paths of mobile elements. (**a**) Path by HiCoDG with SPG; (**b**) Path by HiCoDG with K-means; (c) Path by mTSP.

Figure 8 plot the movement trajectories (*i.e.*, movement paths) for MEs generated by HiCoDG with SPG and K-means algorithms, and mTSP, respectively. The length of each path (*i.e.*, the distance that each ME travels to finish one round) and total length of those paths are presented in Table 3. Note that in Table 3, *ME*_1_ and *ME_i_*(*i* = 2, …, 5) in mTSP correspond to *MR* and *MC_i_*(*i* = 1, …, 4) in HiCoDG, respectively. More specifically, as shown in Figure 8 and Table 3, under mTSP, the difference between the longest and shortest paths for MEs is higher than those of HiCoDG. In other words, under mTSP, an ME may travel a longer distance than others, which results in unbalanced energy consumption. Moreover, under mTSP, the total path length of all MEs is much longer than the total length in HiCoDG.

In addition, as shown in Figure 8 and Table 3, under HiCoDG, when the SPG grouping algorithm is used, the path length of MR is shorter than when using K-means algorithm. The reason is that when the SPG algorithm is used, the path of MR includes POIs which are closer to the sink than when using the K-means algorithm. Note that due to the nature of the K-means algorithm, groups are formed such that POIs in a group are closed to each other without consideration of the sink position. As a result, all POIs in a group can be distant from the sink. In other words, when the K-means algorithm is used, the path of MR may consist of POIs that are distant from the sink, which leads to a longer path length. Moreover, as shown in Table 3, the maximum path length of MCs in K-means algorithm is longer than that of the SPG algorithm. This is possible because a large number of POIs that are close to each other can be in a group when K-means algorithm is used.

Recall that in HiCoDG, along the optimal paths as shown in Figure 8, the MR and MCs cooperate with each other to collect and relay the data from POIs to the sink. More specifically, under IS, the MR and MCs cooperate in a way that each MC periodically drops off the collected data to the MP, and the MR periodically visits the MP and relays the data to the sink. On the other hand, under CMS, a higher level of cooperation is performed. The movement speeds of the MR and MCs are determined such that the MR can periodically meet MCs at MPs to directly receive the data from MCs.

Now we analyze the effects of movement speed of MEs on the performance metrics. The maximum speed for mobile elements varies from 5 to 10, 20, 25, 30 m/s. Recall that, in CMS, the speed of the MEs is determined by using the maximum speed and the path length of the MEs, *i.e.*, the actual movement speeds of the MEs are below the maximum speed. In order to compare the data-gathering latency between CMS and IS that have the same energy consumption for MEs' movements, under IS, MEs always move at the average speed of MEs under CMS for collecting the data. Note that under CMS, when the SPG algorithm is used, the average speed of MEs is higher than when using the K-means algorithm. In this work, in order to favor mTSP in terms of data-gathering latency, we make MEs under mTSP move at the average speed of MEs under CMS with the SPG algorithm.

**Figure 9.**
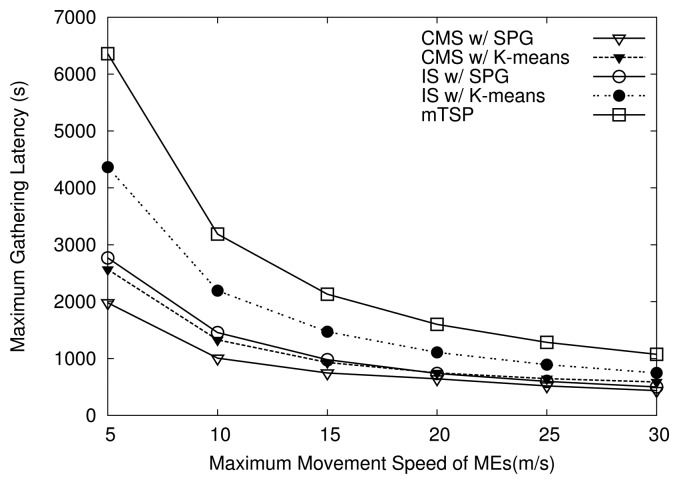
Effects of movement speed on maximum data-gathering latency.

Figure 9 shows the maximum data-gathering latency of the schemes over variations in maximum speed of the MEs. As shown in Figure 9, four variants of HiCoDG always exhibit a lower maximum latency than mTSP over variations in the maximum speed of MEs. For example, when the maximum speed is 20 m/s, the maximum latency of mTSP is about 1600 s, while the maximum latency of HiCoDG schemes is less than 1100 s. The reason is that in mTSP, every ME has to return to the sink at the end of a data-gathering tour, which results in high latency. Moreover, in mTSP, the optimal trajectories for MEs are unbalanced and an ME travels a long path compared to others. On the other hand, in HiCoDG, MCs travel only in the region assigned to them and do not need to return to the sink.

In addition, as shown in Figure 9, in both CMS and IS, when the SPG algorithm is used for grouping, the maximum gathering latency is always lower than when using the K-means algorithm. The reason is that when K-means is used, the path length of the MR is longer than when using the SPG algorithm. Moreover, as shown in Table 3, under K-means, the maximum path length of MCs is longer than that under the SPG algorithm. Note that when the path length of an MC is longer, the time duration from time the packet is generated to time the MC brings it to the MP also becomes longer. As a result, the packet has higher maximum latency when the K-means algorithm is used for grouping.

**Figure 10.**
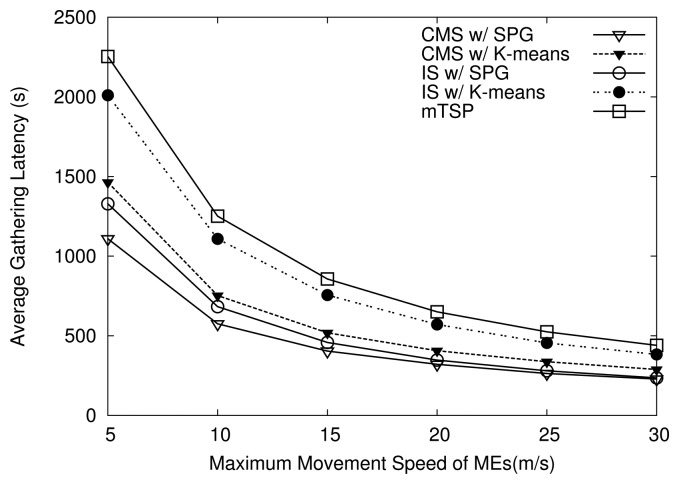
Effects of movement speed on average data-gathering latency.

Figure 10 compares the average data-gathering latency of the algorithms. As shown in Figure 10, the average latency of algorithm are inversely proportional to the maximum speed of MEs, which agrees with the relationship between traveling time and the movement speed on a given distance. Moreover, all variants of HiCoDG have a lower average gathering latency than mTSP. In particular, CMS with SPG consistently shows the lowest latency compared to others. For example, when the maximum speed of MEs is 10 m/s, the average latency of mTSP is 54% higher than the latency of CMS with SPG. In Figure 10, we also see that when the maximum speed of MEs becomes very high, the average latency tends converge to a certain value, which depends on the sojourn time of MEs at POIs and the sink for data transfer as well as the movement speed of MEs.

In addition, as shown in Figure 10, when the same grouping algorithm is used, CMS has a lower average latency than IS. The gap between latency values of CMS and IS tends to decrease as the maximum speed of MEs grows. The reason of lower latency in CMS compared to IS is that, under IS, due the lack of cooperation between the MR and MCs, the packets may need wait for a long time at MPs before the MR arrives at MPs and picks them up. On the other hand, under CMS, the waiting time of the packets at MPs is zero. Moreover, as shown in Figure 10, when the K-means algorithm is used, the latency gap between CMS and IS is higher than when using the SPG algorithm. For example, at the maximum speed of 10 m/s, under K-means algorithm, the latency of CMS is about 32% lower than that of IS, while it is about 16% under SPG algorithm.

**Figure 11.**
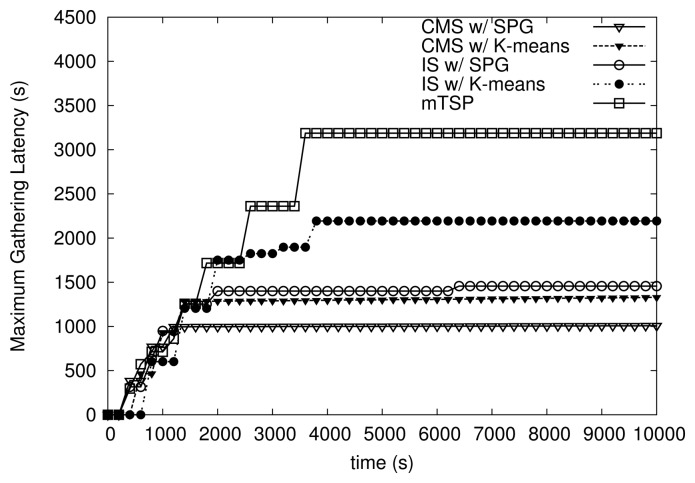
Maximum data-gathering latency over time.

**Figure 12.**
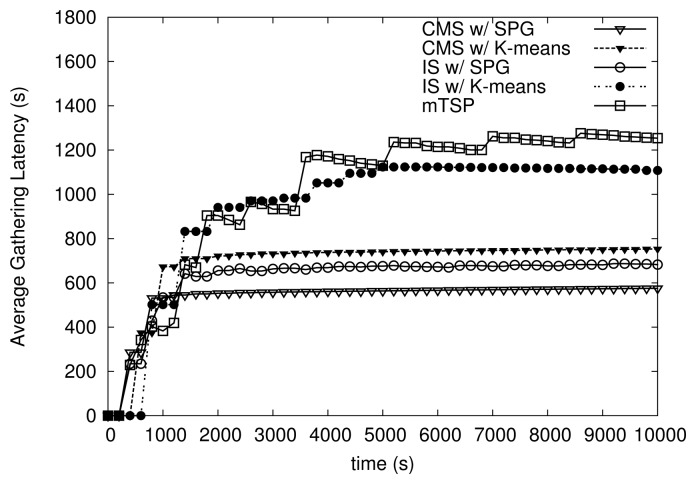
Average data-gathering latency over time.

Figures 11 and 12 show the variation of maximum and average latency of algorithms over time. The maximum speed of MEs is set to 10 m/s. Note that at several time points around time 0, the latency is zero since no packets are collected at the sink. As shown in Figures 11 and 12, all variants of HiCoDG exhibit more stable latency (*i.e.*, maximum and average latency) than mTSP over time. In particular, CMS shows the most consistent latency among algorithms. Recall that in CMS, the initial positions of MCs are determined such that each MC can meet the MR at MP. Thus, at the first round, the MC may start from a middle point of its path, and send the smaller number of packets with a low latency to the MR at the first meeting at MP. Thus, as shown in Figures 11 and 12, at time before 1000 s, CMS has a lower latency than at time after 1000 s.

In addition, note that under IS, due to the lack of movement cooperation among the MR and MCs, the number of packets collected and latency are different over the rounds. Thus, IS shows less consistent latency than CMS. Moreover, as shown in Figure 12, the average latency of mTSP is fluctuating due to the unbalanced trajectories of MEs. From results shown in Figures 11 and 12, we see that variants of HiCoDG achieve not only lower but also more consistent latency than mTSP.

**Figure 13.**
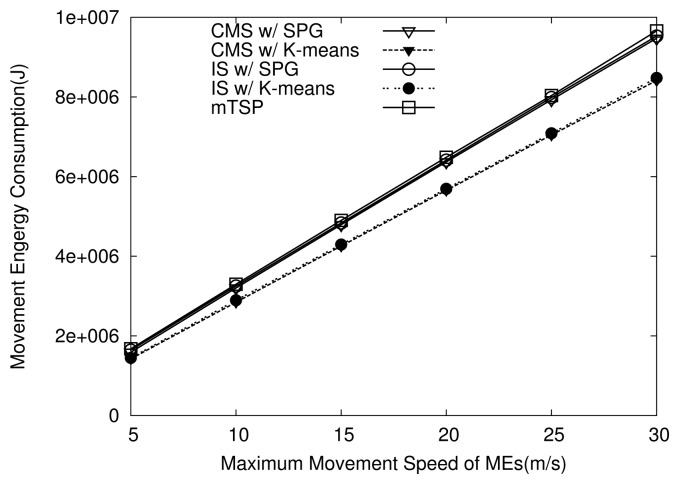
Effects of movement speed on energy consumption.

Figure 13 plots the energy consumed by movements of MEs as their maximum speed increases. As shown in Figure 13, when the same grouping algorithm is used, movement energy consumption under the CMS and IS schemes is similar to each other because they have the same average movement speed of MEs. In addition, in both CMS and IS, the SPG algorithm results in higher energy consumption than the K-means algorithm as shown in Figure 13. This is because when the SPG algorithm is used, the average speed of the MEs under CMS is higher than when using K-means algorithm. Note that mTSP shows similar energy consumption to CMS with SPG because MEs under mTSP move at the average speed of MEs in CMS with SPG.

**Figure 14.**
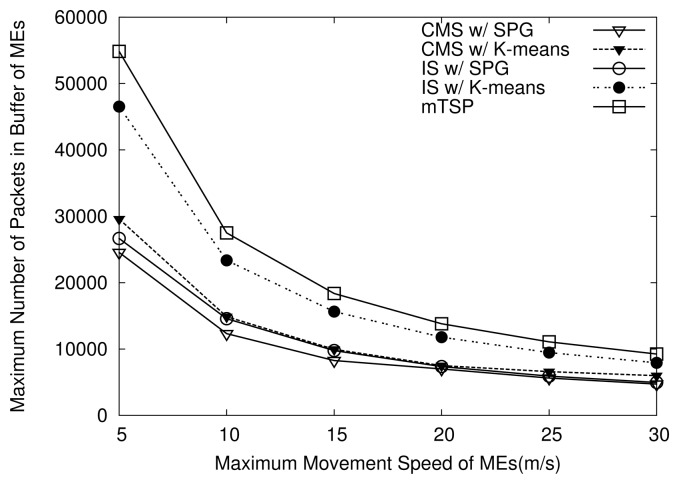
Effects of movement speed on maximum number of packets in buffer of MEs.

Figure 14 shows the maximum number of packets stored in the buffer of MEs when their maximum movement speed varies. As shown in Figure 14, when the speed of MEs increases, the maximum number of packets in the buffer of MEs decreases since the number of packets that are collected by MEs in one round decreases. Moreover, mTSP always exhibits a highest maximum number packets in buffer than others since, under mTSP, an ME travels on a much longer tour compared to others. Note that the maximum number of packets in the buffer of MEs indicates a large size of buffer is required for MEs. Thus, those results imply that mTSP requires a higher capacity buffer for MEs compared to HiCoDG, which leads to the increase of the system cost.

In addition, as shown in Figure 14, when the same grouping algorithm is used, IS always shows a higher maximum number of packets that are stored in the buffer of MEs than CMS does. In both CMS and IS, the MR always has the largest number of packets in the buffer since it brings the data collected by all MCs to the sink at each round. Note that, under IS, the MR might receive the data collected during several rounds of MCs at MPs. On the other hand, under CMS, the MR always receives the data collected in one round of MCs. As a result, under IS, the maximum number of packets stored in the buffer is higher.

Recall that IS requires not only a larger buffer size for MEs but also the data storage in MPs. In order to estimate the capacity of data storage required for MPs in IS, we also collect the maximum number of packets stored in the buffer of MPs. Table 4 shows the maximum number of packets stored in MPs under IS with SPG and K-means algorithms over variations in maximum speed of MEs. When the path lengths of MC and MR are longer, a larger number of packets will be stored in the buffer of the MP waiting for the MR. Thus, as shown in Table 4, when the K-means algorithm is used, the maximum number of packets stored in MPs is larger than when using the SPG algorithm.

In summary, those results imply that on given movement energy consumption, CMS can achieve lower data-gathering latency than IS. In addition, the SPG algorithm results in lower latency but higher energy consumption than the K-means algorithm. Also note that by scheduling the movements of the MEs, the need of the data storage at MPs can be avoided. In other words, lower and more consistent data-gathering latency than the existing approach (e.g., mTSP) can be achieved by using cooperative movement among MEs without the need for special special hardware.

## 6. Concluding Remarks

In this paper, we have proposed a new hierarchical and cooperative data-gathering (HiCoDG) scheme that enables multiple mobile elements to cooperate to collect and relay data. In HiCoDG, there are two types of mobile elements: mobile collectors (MCs) and a mobile relay (MR). MCs collect the data from sensors, and the MR gathers the data from the MCs, delivering them to the sink. An ILP optimization problem has been formulated to find the optimal trajectories for the MCs and the MR with the objective of minimizing energy consumption. Also, we have proposed a cooperative movement scheduling algorithm to determine the optimal movement speeds for MCs and the MR. Simulations have been conducted to compare the performance of HiCoDG with the mTSP-based approach.

For future work, in order to reduce the execution time for finding the trajectories for the MR and MCs, we plan to design a solution method where an approximation algorithm for TSP and a greedy algorithm are applied to find the trajectories for the MC and MR, respectively. We also intend to extend HiCoDG to take into consideration the problem of the limited buffer size of sensors as well as MEs when scheduling the movements for the MEs such that the buffer overflow at sensors and MEs can be avoided.

## Figures and Tables

**Table 1.** Path length configurations.

**Mobile Elements**	**Path Length (m)**	

	**Configuration 1**	**Configuration 2**
*MR*	2935	3844
*MC*_1_	1222	1750
*MC*_2_	2897	3277
*MC*_3_	1999	3320
*MC*_4_	2589	1946

**Table 2.** Movement energy consumption.

**Maximum Speed (m/s)**	**Energy Consumption (10^4^J)**	

	**Configuration 1**	**Configuration 2**

	**CMS & IS**	**CMS & IS**
5	49.2	45.6
10	98.4	91.2
15	147.6	136.9
20	196.8	182.5
25	246.1	228.2
30	295.2	273.7

**Table 3.** Path length of Mobile Elements.

**Mobile Elements**	**Path Length (m)**		

	**HiCoDG w/SPG**	**HiCoDG w/K-Means**	**mTSP**
*ME*_1_ (*MR*)	2459	4172	2346
*ME*_2_ (*MC*_1_)	2873	3680	5643
*ME*_3_ (*MC*_2_)	3805	1665	13,534
*ME*_4_ (*MC*_3_)	3616	4600	4526
*ME*_5_ (*MC*_4_)	2305	1971	9909

Total distance	15,058	16,088	35,958

**Table 4.** Maximum number of packet in buffer of MPs.

**Maximum Speed (m/s)**	**Number of Packets**	

	**IS w/SPG**	**IS w/K-Means**
5	11,266	17,056
10	5668	8568
15	3800	5740
20	2867	4323
25	2308	3475
30	1934	2909

## References

[1] Yick J., Mukherjee B., Ghosal D. (2008). Wireless Sensor Network Survey. Comput. Netw..

[2] Wu X., Chen G., Das S. (2008). Avoiding Energy Holes in Wireless Sensor Networks with Nonuniform Node Distribution. IEEE Trans. Parallel Distrib. Syst..

[3] Di Francesco M., Das S.K., Anastasi G. (2011). Data Collection in Wireless Sensor Networks with Mobile Elements: A Survey. ACM Trans. Sens. Netw..

[4] Le D.V., Oh H., Yoon S. A Novel Hierarchical Cooperative Data Gathering Architecture Using Multiple Mobile Elements.

[5] Wang Z., Basagni S., Melachrinoudis E., Petrioli C. Exploiting Sink Mobility for Maximizing Sensor Networks Lifetime.

[6] Ma M., Yang Y. (2007). SenCar: An Energy-Efficient Data Gathering Mechanism for Large-Scale Multihop Sensor Networks. IEEE Trans. Parallel Distrib. Syst..

[7] Yuan B., Orlowska M., Sadiq S. (2007). On the Optimal Robot Routing Problem in Wireless Sensor Networks. IEEE Trans. Knowl. Data Eng..

[8] Gu Y., Bozdag D., Ekici E., Ozguner F., Lee C.G. Partitioning Based Mobile Element Scheduling in Wireless Sensor Networks.

[9] Somasundara A.A., Ramamoorthy A., Srivastava M.B. Mobile Element Scheduling for Efficient Data Collection in Wireless Sensor Networks with Dynamic Deadlines.

[10] Guo S., Wang C., Yang Y. Mobile Data Gathering with Wireless Energy Replenishment in Rechargeable Sensor Networks.

[11] Kumar A.K., Sivalingam K.M., Kumar A. (2013). On Reducing Delay in Mobile Data Collection Based Wireless Sensor Networks. Wirel. Netw..

[12] Sugihara R., Gupta R. (2010). Optimal Speed Control of Mobile Node for Data Collection in Sensor Networks. IEEE Trans. Mob. Comput..

[13] Gao S., Zhang H., Das S.K. (2011). Efficient Data Collection in Wireless Sensor Networks with Path-Constrained Mobile Sinks. IEEE Trans. Mob. Comput..

[14] Zhao M., Ma M., Yang Y. (2011). Efficient Data Gathering with Mobile Collectors and Space-Division Multiple Access Technique in Wireless Sensor Networks. IEEE Trans. Comput..

[15] Jea D., Somasundara A., Srivastava M. (2005). Multiple Controlled Mobile Elements (Data Mules) for Data Collection in Sensor Networks. Distributed Computing in Sensor Systems.

[16] Kim D., Abay B., Uma R.N., Wu W., Wang W., Tokuta A. Minimizing Data Collection Latency in Wireless Sensor Network with Multiple Mobile Elements.

[17] Gu Z., Hua Q.S., Wang Y., Lau F. Reducing Information Gathering Latency Through Mobile Aerial Sensor Network.

[18] He L., Pan J., Xu J. Analysis on Data Collection with Multiple Mobile Elements in Wireless Sensor Networks.

[19] He L., Pan J., Xu J. Reducing Data Collection Latency in Wireless Sensor Networks with Mobile Elements.

[20] He L., Pan J., Xu J. (2013). A Progressive Approach to Reducing Data Collection Latency in Wireless Sensor Networks with Mobile Elements. IEEE Trans. Mob. Comput..

[21] Aslanyan H., Leone P., Rolim J. Data Propagation with Guaranteed Delivery for Mobile Networks.

[22] Cai C., Yang C., Zhu Q., Liang Y. Collision Avoidance in Multi-Robot Systems.

[23] Hennes D., Claes D., Meeussen W., Tuyls K. Multi-robot Collision Avoidance with Localization Uncertainty.

[24] Tselikis C., Mitropoulos S., Komninos N., Douligeris C. (2012). Degree-Based Clustering Algorithms for Wireless Ad Hoc Networks Under Attack. IEEE Commun. Lett..

[25] Peng W., Edwards D. K-Means Like Minimum Mean Distance Algorithm for wireless sensor networks.

[26] Pataki G. (2003). Teaching Integer Programming Formulations Using The Traveling Salesman Problem. SIAM Rev..

[27] Kara I., Bektas T. (2006). Integer linear Programming Formulations of Multiple Salesman Problems and Its Variations. Eur. J. Oper. Res..

[28] Kara I., Bektas T. Integer Linear Programming Formulation of the Generalized Vehicle Routing Problem.

[29] Papadimitriou C.H. (1977). The Euclidean Travelling Salesman Problem is NP-complete. Theor. Comput. Sci..

[30] Le D.V., Oh H., Yoon S. (2013). RoCoMAR: Robots' Controllable Mobility Aided Routing and Relay Architecture for Mobile Sensor Networks. Sensors.

[31] GLPK (GNU Linear Programming Kit). https://www.gnu.org/software/glpk/.

[32] Rahimi M., Shah H., Sukhatme G., Heideman J., Estrin D. Studying the Feasibility of Energy Harvesting in a Mobile Sensor Network.

